# Carvedilol in the Treatment of Portal Hypertension

**DOI:** 10.4103/1319-3767.77251

**Published:** 2011

**Authors:** Hamdan Al-Ghamdi

**Affiliations:** Department of Hepatobiliary Sciences, King Abdulaziz Medical City, Riyadh, Saudi Arabia

**Keywords:** Band ligation, bleeding, carvedilol, portal hypertension, treatment, varices

## Abstract

Variceal bleeding is a major event in the natural history of end-stage liver disease with a subsequent high mortality rate. Non-selective β-blockers are currently the drugs of choice for preventing first variceal bleeding. Endoscopic rubber band ligation of high risk varices features as a first line option if cirrhotic patients cannot tolerate β-blockers. Despite adequate β-blockade, some patients may still present with variceal bleeding. The effect of carvedilol, a non-selective β and α-1 receptor-blocker, on lowering portal pressure has been investigated in several clinical trials and found to be superior to propranolol in both acute and chronic hemodynamic studies. Recently, carvedilol has also been compared with band ligation for primary prophylaxis against variceal bleeding with equivalent results to band ligation. Patient tolerance to carvedilol in advanced liver disease remains a source of concern. This review examines the place of carvedilol as an alternative to the currently recommended pharmacological therapy in prophylaxis against variceal bleeding.

## TREATMENT OVERVIEW OF PORTAL HYPERTENSION

Hepatic hemodynamic studies enable us to indirectly estimate the degree of portal hypertension in cirrhotic patients. Clinically significant portal hypertension is defined as hepatic venous pressure gradient (HVPG) greater than 10 mmHg.[1] It is estimated that for every 1 mmHg increase in HVPG above a threshold level of 10 mmHg, an 11% increase in the risk of clinical decompensation could be expected.[2]

The clinical course of cirrhosis, commonly identified as compensated and decompensated forms, has recently been characterized into four prognostic stages.[3] Stage 1 is characterized by the absence of esophageal varices and ascites with an annual mortality rate of 1%; stage 2 is characterized by the appearance of esophageal varices without ascites and has an annual mortality rate of 3.4%, and stage 3 is characterized by ascites with or without esophageal varices and has an annual mortality rate of 20%. Finally, stage 4 is characterized by variceal bleeding, with or without ascites, and has 1-year mortality rate of 57%, with almost half of this occurring in the first 6 weeks of index bleeding. An increased portal pressure in cirrhotic patients plays a fundamental role in the development and rupture of varices and a HVPG over 12 mmHg was found to be a strong predictor for esophageal variceal bleeding.[4]

Given the significant mortality associated with variceal bleeding, and that varices may be present in a majority of cirrhotics, clinical trials dealing with portal pressure reduction have channeled their efforts into three main strategies. The first strategy (pre-primary prophylaxis) dealt with prevention of gastroesophageal varices development by using non-selective β-blockers. This strategy was eventually found to be ineffective in preventing development of varices.[5] The second strategy (primary prophylaxis) involved the prevention of variceal bleeding in patients who experienced no previous bleeding where high risk varices such as large varices (larger than 5 mm) or varices with red signs (red wales, cherry red spots, or hematocystic spots) had been identified at endoscopy. Non-selective β-blockers (propranolol and nadolol) demonstrated superiority to placebo in preventing variceal bleeding.[6] Endoscopic rubber band ligations of high risk varices were compared head-to-head with non-selective β-blockers in several randomized control studies as primary prophylaxis strategy. Several meta-analyses[7–10] reported a small but significantly lower incidence of first variceal bleeding but no survival benefits were found in favor of band ligation. The third strategy (secondary prophylaxis) entails using non-selective β-blockers alone or in combination with endoscopic variceal band ligations/sclerotherapy for preventing rebleeding. The current evidence seems to favor combination therapy (non-selective β-blockers and variceal band ligation) over band ligation alone.[11,12]

Salvage invasive techniques such as transjugular intrahepatic portosystemic shunt (TIPS) and surgical devascularization/shunting are typically reserved for refractory variceal bleeding when appropriately indicated.

## PHARMACOLOGICAL ASPECTS

Non-selective β-blockers exert their effects via blockade of both β1 and β2 receptors. The β1 receptors are located in the myocardium and their blockade results in a decrease in cardiac contractility and output. In the other hand, blockade of β2 receptors located in the splanchnic (mesenteric) vascular bed results in vasoconstriction due to unopposed activity of α1 adrenergic receptors with a net effect of reduction in portal blood inflow.

The intrahepatic vascular resistance represents another essential component in development and progress of portal hypertension in cirrhotic. It is caused by morphological changes in the hepatic microcirculation architecture due to pathological changes associated with cirrhosis. It is estimated that up to 40% of this intrahepatic vascular resistance is functionally reversible.[13] This reversible part is located in the contractile elements of the hepatic vascular bed. It pharmacologically represents another major site to modulate portal hypertension.

The α1 adrenergic receptors are distributed in both the systemic and splanchnic vascular smooth muscles and other sites such as smooth muscles of the genitourinary tract. Pharmacological antagonism of α1 adrenergic receptors would lead to a reduction in the intrahepatic vascular tone. Therefore, the addition of α1 blockers to non-selective β-blockers may attenuate portal pressure. In one trial, propranolol was combined with prazosin (n = 28), a selective α1 blocker, and compared to propranolol plus isosorbide-5-mononitrate (n = 28) for short-term use over 3 months.[14] The former combination showed a greater reduction in HVPG (24% vs 16%) but more hypotension events and less tolerance.

Carvedilol[15,16] is a racemic mixture that has potent non-selective β-receptors and weak α-1 receptors blocking activity. It is two to four times more potent than propranolol as a β-receptor blocking drug. It has two enantiomeric forms, *R* (+) and *S* (–). The *S* (–) enantiomer is mainly responsible for the β-blocking effect of carvedilol, whereas both *R* (+) and *S* (–) contribute to the a1-blockade. It is rapidly absorbed following oral administration with a low absolute bioavailability, approximating to 25%. It has a rapid onset of action of 1-2 h. It undergoes extensive hepatic first-pass metabolism with plasma levels of *R* (+)-carvedilol two to fourfold higher than *S* (–)-carvedilol in healthy persons. The major P450 enzymes responsible for the metabolism of both *R* (+) and *S* (–) carvedilol in human liver microsomes are CYP2D6 and CYP2C9, and to a lesser extent CYP3A4, CYP2C19, and CYP2E1. The elimination half-life of carvedilol is 6-10 h. Drugs that inhibit CYP450 2D6 activity such as sertraline may increase plasma concentrations of R-carvedilol, in turn augmenting the systemic hypotensive effect of carvedilol. Drug excretion is mainly biliary and into feces, and renal elimination is minor requiring no dose adjustment in kidney impairment.

Carvedilol is available in various immediate release (used as once or twice daily) and extended release formulations (used as once daily). The drug is FDA approved for management of essential hypertension, congestive heart failure, and left ventricular dysfunction following myocardial infarction.

## CLINICAL TRIALS IN CARVEDILOL

Due to its unique mechanism of action, carvedilol was compared to propranolol in several clinical trials.[17–19,22] In the portal hemodynamic studies that evaluated the risk of variceal bleeding, responders to non-selective β-blockers were recognized if their HVPG dropped below 12 mmHg or by more than 20% from baseline.

Clinical trials evaluating the acute hemodynamic effects of carvedilol at a dose of 25 mg on portal pressure showed a reduction in HVPG by 17–27% from baseline measurements[17–20] [Table 1]. In these trials, the incidence of systemic hypotension was significantly higher in the carvedilol group. On the other hand, hemodynamic effects of chronic administration of carvedilol were reported in several trials[19–23] using variable dosages between 12.5 and 50 mg/day. Banares *et al*,[22] reported the longest follow-up trial of 11.1 ± 4.1 weeks in 51 cirrhotic patients (26/carvidelol, 25/propranolol). The carvedilol doses were administrated at 12.5-50 mg (mean 31 ± 4 mg/d) starting at 6.25 mg and titrated up every 4 days according to blood pressure and heart rate. Chronic carvedilol administration resulted in 58% hemodynamic response rate compared to 23% response rate in the propranolol group. The mean arterial blood pressure (MAP) and systemic vascular resistance (SVR) weres not statistically different between baseline and end-of-study measurements in the carvedilol group. In acute carvedilol administration studies, there was a noticeable decrease in MAP and SVR. However, in this study carvedilol did not decrease SVR, but had a mild decrease in MAP. There was an increase in plasma volume and body weight that were statistically significant in the carvedilol group as observed in Child-Pugh class B and C patients. This required dose adjustment of diuretics. There were no changes in glomerular filtration rate (GFR) or urinary sodium excretion in either group. Carvedilol caused a greater decrease in HVPG in patients with Child-Pugh classes B and C, despite requiring lower doses, than Child-Pugh class A cirrhotic patients. Adverse events (orthostatic hypotension, encephalopathy, and discontinuation of the treatment) were not significantly different between the two treatment groups. The discontinuation rate of carvedilol was 8% as compared to 12% in the propranolol arm.

**Table 1:** Studies reporting on the effect of carvedilol in lowering hepatic venous pressure gradient

Author (year)	Number of patients		Study type	Hepatic venous pressure gradient (HVPG)		
	Propranolol (n)	Carvedilol (n)		Baseline (mmHg)	Post-propranolol (% difference from baseline)	Post-carvedilol (% difference from baseline)
Forrest *et al*,[20] (1996)	-	16	Acute	16.7 ± 0.9	-	13.6 ± 1 (-18)
Banares *et al*,[17] (1999)	14		Acute	Carvedilol 19.5 ± 1.3	-	15.4 ±1 (-21)
		14		Propranolol 20.4 ± 1.1	17.7 ± 0.8 (-13)	-
Tripathi *et al*,[21] (2002)	-	19	Acute	16.37 ± 2.14	-	12.56 ± 3.91 (-23)
			Chronic	16.37 ± 0.71	-	9.27 ± 1.40 (-43)
De *et al*,[19] (2002)	18	18	Acute	Carvedilol 19 ± 3.79	-	13.7 ± 5.94 (-27)
				Propranolol 16.6± 3.96	12.9 ± 6.02 (-22)	-
			Chronic	Carvedilol 19 ± 3.79	-	13.6 ± 5.42 (-28)
				Propranolol 16.6± 3.96	13.10 ± 5.31 (-21)	-
Banares *et al*,[22] (2002)	25	26	Chronic	Carvedilol 19.0 ± 1.1	-	15.2 ± 0.8 (-20)
				Propranolol 20.3 ± 0.9	17.6 ± 0.7 (-13)	-
Lin *et al*,[18] (2004)	11	11	Acute	Propranolol + ISM 17.6 ± 1.2	14.8 ± 0.9 (-15)	-
				Carvedilol 18.9 ± 1.8	-	15.6 ± 1.9 (-17)
Bruha *et al*,[23] (2006)	-	36	Chronic	17.7 ± 3.8	-	14.9 ± 4.8 (-15)

ISM=isosorbide-5-mononitrate, Figures in parentheses are in percentage

Chronic carvedilol administration at lower doses of 12.5 mg was studied in two clinical trials.[19,21] Both trials reported a reduction in HVPG by 23–43% from baseline measurements without a significant effect on MAP.

In the first and only randomized controlled trial comparing carvedilol with variceal band ligation for primary prophylaxis against variceal bleeding,[24] 152 cirrhotic patients with grade II or larger esophageal varices were randomized to either carvedilol 12.5 mg once daily (77 patients) or variceal band ligation (75 patients) every 2 weeks until eradication. On intention-to-treat analysis, carvedilol achieved lower rates of the first variceal bleed 10% versus 23% without differences in the overall mortality (35% vs 37%) or bleeding-related mortality (3% vs 1%). In this study, 15% of patients discontinued carvedilol due to intolerance. On per protocol analysis, there was however no difference between both groups in terms of first variceal bleeding, overall mortality, or bleeding-related mortality. There was no change in mean arterial pressure or serum creatinine in the carvedilol arm. Worsening of ascites was not different between carvedilol (18%) and band ligation (21%) arms.

It should be noted that in these clinical trials, the predominant liver dysfunction was Child-Pugh class A. Cirrhotic class B or C patients were more likely to suffer adverse effects, particularly when doses higher than 12.5 mg were used. It is also worth addressing that lower doses of carvedilol at 12.5 mg showed a comparable portal hemodynamic effect when administrated chronically as reported from at least two studies.[19,21] Titration of carvedilol dose is perhaps the best strategy. Precipitating or worsening of hypotension, ascites, or renal function was noted in some but not all trials, particularly when doses of 25 mg or higher were administrated.

Given the powerful effect of carvedilol on portal hemodynamics, the question remains whether carvedilol should be the drug of choice in patients with varices who have non-cirrhotic portal hypertension? As of yet, this issue has not been addressed yet in this cohort of patients who may tolerate higher doses of carvedilol. Secondly, another question that remains unanswered is whether carvedilol should replace non-selective β-blockers in Child-Pugh class A cirrhotic patients presenting with variceal bleeding despite adequate β-blockade? Finally, despite of the promising results reported in these trials, larger randomized, controlled clinical trials comparing carvedilol with other non-selective β-blockers, or further head-to-head trials comparing carvedilol to band ligation must be conducted. Adequately powered trials with better patient selection, well-defined clinical end points, careful assessment of adverse events, and sufficient follow-up are required before we can advance carvedilol into an evidence-based treatment algorithm of portal hypertension.
